# Use of Hexapod External Fixation in Limb Lengthening in Patients with Disproportionate Short Stature: A Systematic Review of the Last 20 Years

**DOI:** 10.3390/jcm14041091

**Published:** 2025-02-08

**Authors:** Gianluca Testa, Michela Marchetti, Marco Sapienza, Martina Ilardo, Sebastiano Mangano, Giuseppe Condorelli, Vito Pavone

**Affiliations:** Department of General Surgery and Medical Surgical Specialties, Section of Orthopaedics, A.O.U. Policlinico Rodolico-San Marco, University of Catania, 95123 Catania, Italy; michelamarchetti99@tiscali.it (M.M.); marcosapienza09@yahoo.it (M.S.); martinailardo52@gmail.com (M.I.); sebymangano@hotmail.com (S.M.); dott.condorelli@libero.it (G.C.); vitopavone@hotmail.com (V.P.)

**Keywords:** lengthening, hexapod external fixation, deformity, systematic review, correction, TSF, pin infections

## Abstract

**Background:** Limb lengthening is a surgical procedure intended to correct discrepancies and deformities in limb length or to enhance limb length for functional or cosmetic reasons. Short stature, often seen as a physical condition, can significantly affect a patient’s quality of life. The advancement of limb lengthening methods, including the creation of hexapod external fixation systems, has heightened the precision and efficacy of these procedures. The Taylor spatial frame (TSF), a form of hexapod external fixator, grants three-dimensional control of bone movement and is increasingly used to rectify deformities and lengthen limbs. This systematic review aims to assess the effectiveness of the hexapod external fixator in limb lengthening and deformity correction compared to other external fixation systems, focusing on outcomes such as achieved lengthening, healing index, complications, and follow-up duration. **Methods**: A structured search was engineered in four crucial search engines (PubMed, Scopus, Web of Science, and Medline) spanning 2004 to 2024. **Results**: The studies included in this review indicate that the average lengthening accomplished with the hexapod fixator ranged from 3 to 5.9 cm, with a healing index between 37 and 68.6 days/cm. The most frequent complications were pin site infections, compartment syndrome, and delayed union. The follow-up duration ranged from 6.8 months to 6 years. These studies also compared the hexapod external fixator with other external fixators, showing that while the TSF allowed more accurate deformity corrections, it often displayed a higher healing index. **Conclusions**: The hexapod external fixator, specifically the TSF, is an effective instrument for limb lengthening and deformity correction in patients with short stature. Although it provides superior three-dimensional control for deformity correction, the healing index and treatment duration can be longer compared to traditional external fixators. Further studies with larger sample sizes and extended follow-up are needed to perfect treatment protocols and thoroughly evaluate the long-term outcomes and complications associated with this technique.

## 1. Introduction

Limb lengthening is a surgical procedure used to correct limb length discrepancies and deformities, or to increase the length of a limb for functional or cosmetic reasons. Short stature is more than a physical condition; it may negatively impact the quality of life for patients [1]. These individuals often require constant assistance in performing daily activities, such as sitting down or standing up from a chair. The aim of limb lengthening is to enhance these patients’ quality of life.

The evolution of limb lengthening techniques has significantly advanced with the development of more precise and effective systems. The Ilizarov method was one of the pioneering techniques, utilizing circular external fixation. This method incorporated a complex system of rings, wires, and screws to stabilize the bone during gradual distraction following a deliberate bone break [2]. However, the advent of hexapod external fixation systems, such as the Taylor spatial frame (TSF)—a circular external fixator that employs a computer software program—has revolutionized limb lengthening procedures [3].

The TSF, introduced by Dr. Charles Taylor in 1994, transformed the understanding and ability to perform indirect reduction and correction of deformities and fractures. It is an external fixator offering three-dimensional control of bone movement. The apparatus employs a combination of rings and bars that, when connected through wires and pins, permit gradual lengthening and correction of deformities. However, employing the TSF necessitates an experienced team of orthopedists capable of accurately handling any corrections [4].

The aim of this review is to assess, based on the literature, the efficacy of the hexapod external fixator compared to other fixators in lengthening patients with short stature and correcting anatomical deformities.

## 2. Materials and Methods

### 2.1. Search Strategy

We conducted a structured search of four search engines from 2004 to 2024: PubMed, Scopus, Web of Science, and Medline. Initially, we used the following words in our literature search: “Lengthening” AND “Short stature” AND “Deformity”, “Hexapod external fixation” OR “Taylor Spatial Frame”. We employed Boolean operators to combine topic words with keywords and search for references in the related literature. Our search adhered to the Preferred Reporting Items for Systematic Review and Meta-Analysis (PRISMA) guidelines for a systematic review of rates.

### 2.2. Inclusion End Exclusion Criteria

The inclusion criteria were as follows: any level of evidence studies; studies written in English; studies with five or more patients; full-text articles; and studies with a methodological index for non-randomized studies (MINORS) quality evaluation score of more than 14 points.

The exclusion criteria were as follows: review articles, case reports, and articles with fewer than five patients; articles written in languages other than English; non-human studies; absence of full-text articles; studies with a MINORS quality evaluation score of 13 points or less.

### 2.3. Quality Evaluation Using the MINORS Checklist

**Figure 1:**
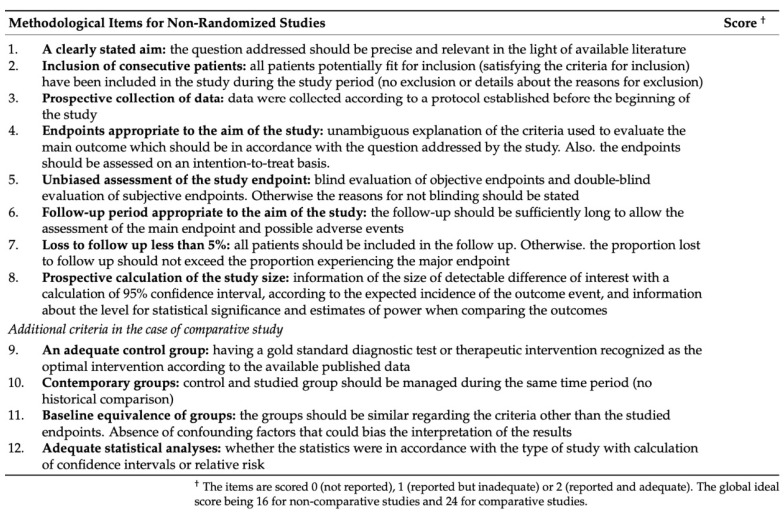
The revised and validated version of MINORS.

The MINORS checklist is used to evaluate the adequacy of items based on eight indicators for non-comparative studies. These include a clearly stated aim; the inclusion of consecutive patients; prospective data collection; endpoints appropriate to the aim of the study; unbiased assessment of the study endpoint; follow-up period suitable for the study’s aim; a loss-to-follow-up of less than 5%; and prospective calculation of the study size. Items are scored as follows: 0 (not reported), 1 (reported but inadequate), or 2 (reported and adequate) [5] (Figure 1).

### 2.4. Data Extraction

**Figure 2:**
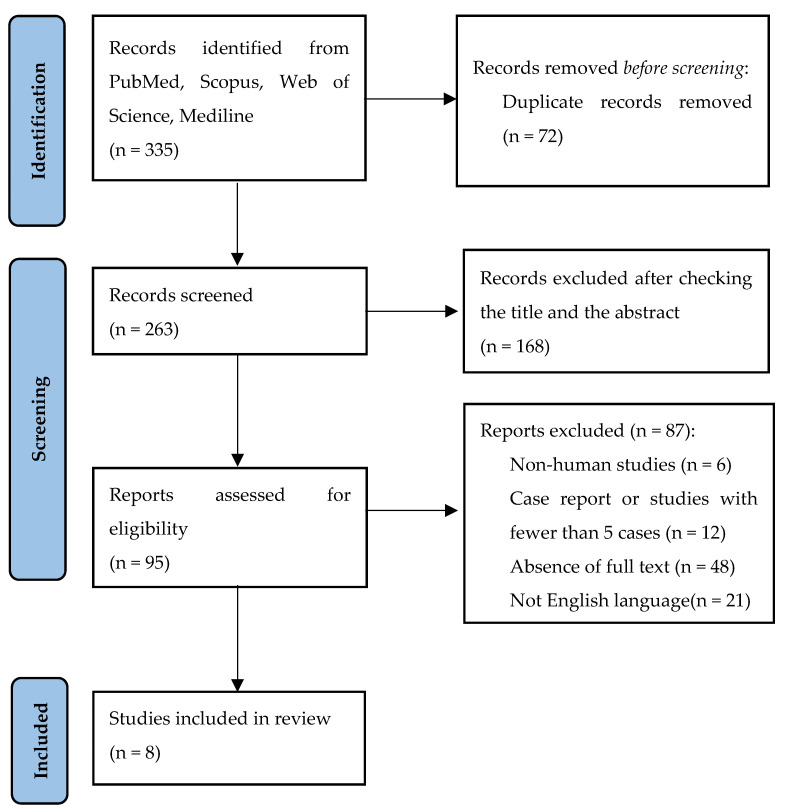
PRISMA 2020 flowchart for the selection and screening method.

Data were extracted using the PRISMA flowchart. The PRISMA flowchart illustrating the selection and screening method is provided in Figure 2.

The data extracted for analysis included the first author and year of publication, the study type, patient count and sex ratio, average age at operation, patients’ clinical characteristics, the type of implant and bone involved, average length increase in cm, healing index (days/cm), follow-up, complications, and limitations of the study.

### 2.5. Statistical Analysis

The statistical data was analyzed using Microsoft Excel 2021^®^. The use of descriptive statistics was sufficient, and there was no need to calculate significance levels.

## 3. Results

### 3.1. Search Results

Our search strategy yielded 335 articles from 2004 to 2024, incorporating the previously reported keywords. After eliminating duplicates through manual screening, we were left with 263 articles. Upon reviewing the titles and abstracts, we discarded 168 articles/papers, leaving us with 95 articles. From these, we excluded non-human studies, case reports, studies involving fewer than five cases, articles where the complete text was unavailable, and articles not written in English. Ultimately, we identified eight articles that met our inclusion and exclusion criteria.

### 3.2. Basic Characteristics of Included Studies

The basic characteristics of the included studies are included in Table 1 [2,4,6,7,8,9,10,11].

Eight studies were identified, including three randomized controlled trials and five cohort studies. These studies divided patients based on etiology, with some comparing the use of hexapod external fixators with other types of external fixators.

Three studies evaluated the application of hexapod external fixation in a specified patient group, specifically focusing on the average length achieved, the average healing rate, and complications.

Five studies compared the results acquired from different groups, separated based on etiology or the type of external fixator used.

### 3.3. Patient Demographics

The studies encompassed a total of 479 patients, with an average age of 14.6 years. The reasons for limb lengthening encompass congenital deformities, post-traumatic deformities, and acquired deformities.

### 3.4. Lengthening Achieved

The lengthening achieved ranged from 3 to 5.9 cm. The healing index is the ratio between the total treatment time (TTT, defined as the number of days from EF implant to removal) and the total lengthening outcome achieved, expressed in days/cm^2^. It ranged from 37 to 68.6 days/cm^2^.

### 3.5. Duration of Follow-Up

The duration of follow-up refers to the time that patients were monitored after the removal of the EF. It is dependent on the patient’s age at the time of the operation, with younger patients requiring a more extended follow-up than older ones. The average follow-up period ranged from 6.8 months to 6 years post-EF removal.

### 3.6. Complications

Most of these studies utilize Paley’s classification to distinguish between problems, obstacles, and true complications. Problems are defined as postoperative difficulties that are completely resolved with nonoperative intervention. Obstacles, on the other hand, are difficulties that necessitate operative intervention but resolve after surgery. True complications are problems that occur intraoperatively and remain unresolved following treatment completion [7]. The most frequent complications include pin site infections, compartment syndrome, and delayed union. Less common complications encompass deep infections and neurovascular injury.

## 4. Discussion

This systematic review evaluated the use of hexapod external fixation (HEF) for limb lengthening in patients with short stature and deformities. The primary focus was on outcomes related to lengthening achieved, healing index, complications, and follow-up duration. After a thorough screening of 335 articles, only eight studies met the inclusion criteria, collectively involving a total of 479 patients. The studies provided invaluable insights into the effectiveness and safety of HEF for treating short stature and deformities of various etiologies.

The lengths achieved in the studies spanned from 3 to 5.9 cm, aligning with prior records that advocate moderate lengthening outcomes with external fixators for deformity correction in pediatric and adolescent ages. The healing index, fluctuating between 37 and 68.6 days/cm, portrays the efficiency of the bone healing procedure during lengthening. Even though the healing index seen in this review is relatively high in contrast to certain other reports on conventional external fixation methods (20–30 days/cm), it fits the span noted for hexapod external fixators, renowned for their precision and multi-plane deformity rectification aptitude. A heightened healing index may serve as a sign of the cases’ complexity, as clients often come with congenital or post-injury deformities that demand not only lengthening but also conspicuous correction of angular deformities [10].

There is no gold standard for the follow-up of patients treated with hexapod external fixation for limb lengthening. An individualized approach and careful management during all phases of treatment are crucial to achieving optimal results and minimizing risks. The frequency of visits depends on the stage of treatment. During the lengthening period, weekly visits are scheduled to ensure that the lengthening process is safe and that there are no signs of infection or misalignment; during the consolidation period, visits may be less frequent, and after the external fixation removal, long-term visits are made to monitor functional recovery, bone integration, and long-term stability. Most of the studies included in this review state that the duration of follow-up post-external fixation removal depends on the age of the patients at the time of surgery. The mean follow-up duration showed significant variability, ranging from 6.8 months to 6 years, reflecting the variability in patient age and the nature of their deformities. Generally, younger patients require a longer follow-up to confirm complete bone healing and monitor for potential long-term complications [2]. Although most of the studies included in this review reported a follow-up duration exceeding one year, several studies featured a shorter follow-up time. This fact limits the assessment of late complications such as joint stiffness or growth disturbances. This fluctuation in follow-up duration might contribute to some inconsistencies in reported complication rates across the various studies.

The complications associated with HEF, as reported in the reviewed studies, were consistent with those observed in earlier studies concerning limb lengthening with external fixators [7]. Pin site infections, compartment syndrome, and delayed union were the most common issues, which were categorized according to Paley’s classification [2]. These complications are well-known in external fixation techniques, largely relating to the mechanical stresses and soft tissue involvement during the distraction process. Though less common complications such as deep infections and neurovascular injuries were also reported, their incidence remains relatively low.

Pin site infections, though common, were typically managed conservatively, and complications such as compartment syndrome and delayed union were addressed with surgical intervention. The hexapod fixator’s capacity to deliver multidimensional correction may contribute to a slightly higher occurrence of these complications. However, it also permits more precise correction of deformities, potentially reducing the requirement for more invasive surgeries in the future. The comparatively low occurrence of severe complications such as neurovascular injury, a concern with other forms of external fixation, demonstrates the safety of the hexapod system compared to other techniques [12].

The magnetic intramedullary nail (MIN) represents a more recent innovation in the limb lengthening field. This device operates via an external magnetic field that stimulates the mechanical expansion of an internal nail, typically inserted into the intramedullary canal of a bone. This system facilitates non-invasive lengthening and deformity correction, with the external device merely controlling the magnetic field that propels the internal expansion [13].

A key advantage of the MIN is its minimal invasiveness compared to the hexapod external fixator [14,15]. The procedure involves a singular surgical incision for nail insertion, eliminating external components protruding from the limb. This reduction in external features diminishes the risk of infection, enhances patient comfort, and delivers a more aesthetic outcome [16]. Additionally, as the nail is internal, external adjustments and cumbersome devices are unnecessary, significantly improving the patient’s quality of life during treatment.

Additionally, the automatic lengthening process, managed by a remote magnetic system, provides greater precision and predictability. The rate and rhythm of lengthening can be adjusted with minimal patient intervention, thereby reducing the frequency of clinical visits and manual adjustments, a common requirement with external fixators.

Nonetheless, despite the distinct advantages posed by the magnetic nail in terms of comfort and convenience, it comes with its own limitations. The most significant challenge is its technical complexity, which requires precise surgical planning. The intramedullary nail may not be suitable for all deformities, particularly those necessitating significant correction in multiple planes. In such situations, external fixators prove to be more versatile. Additionally, the cost of the MIN is relatively high, and its availability may be limited within various healthcare systems, posing a hindrance to its accessibility.

Lengthening over an intramedullary device in conjunction with an external fixator has been examined in the context of lower limbs. Paley et al. demonstrated that the outcomes of lengthening over an intramedullary nail, when combined with an external fixator, were superior in a population of young adults compared to using an external fixator alone [12].

Several studies have compared the use of the hexapod external fixator with other types of external fixators, such as Ilizarov frames. The results indicated that the hexapod system provided more precise correction in three-dimensional space and was associated with slightly improved functional outcomes. However, the increased complexity of using the hexapod frame may contribute to extended treatment time and a higher healing index observed in some studies. Therefore, the decision to use the hexapod external fixator should depend on the patient’s specific needs, including the severity and complexity of the deformity, as well as the patient’s age and potential for future growth.

Despite the value provided by this systematic review, several constraints need acknowledgment. The relatively small quantity of studies included (n = 8) alongside the variation in study design, patient demographics, and follow-up durations hinders firm conclusions regarding the long-term effectiveness and safety of the hexapod external fixator for limb lengthening across all patient groups. Also, the restriction to English language studies might introduce a language bias, thus restricting the generalizability of the conclusions. Moreover, the studies considered in this analysis varied in quality scores per the MINORS checklist, which might impact the reliability of particular findings. To the best of our knowledge, this cutoff is not standardized and reflects a subjective decision. This could introduce significant bias. Future research with more substantial sample sizes, extended follow-up durations, and standardized methodologies is necessary for a more precise understanding of the long-term effects correlated with HEF.

## 5. Conclusions

In conclusion, the hexapod external fixator seems to be an effective and relatively safe method for limb lengthening in patients with short stature and deformities. Good outcomes were observed in terms of achieved lengthening and typical complications for external fixation techniques. While the healing index is slightly elevated in comparison to traditional external fixators, the precision and capability to correct multi-planar deformities make the hexapod a beneficial option for specific patient groups. However, due to the heterogeneity of the studies and the lack of quantitative data, it is impossible to do a meta-analysis. Further high-quality studies involving larger sample sizes and longer follow-up periods are required to confirm these outcomes and refine treatment protocols using this technique.

## Figures and Tables

**Table 1:** Results of selected studies. CT (clinical trials); RCT (randomized controlled trials).

Author and Study Year	Type of Study	Number of Patients (Sex Ratio)	Age (Years)	Patients	Bone	Implant	Increased Length (cm)	Healing Index (Days/cm)	Follow-Up	Complications	Limitations
Adrien Roy et al., 2020 [6]	RCT	44 (20 F/24M)	11.4	Congenital deformity (86%)	23 Femurs; 35 Tibias	Hexapod external fixation (all)	5.9 +/− 2.7	37 +/− 16	6.8 months	8 early bone unions; 6 overly fast lengthening; 2 flexion deformities; 2 misalignments during the correction; 1 fracture	Small sample size
D. Dammerer et al., 2011 [2]	CT	80	16.4		Tibias; Femurs	Hexapod external fixation (A); Ilizarov fixator (B); Orthofix dynamic axial fixator (C)	3 (A); 4 (B); 4 (C)	68.7 (A); 52.9 (B); 55 (C)		pin site problems and rate of reduced knee motion for ring fixators (A);	Small sample size; number of factors that might have biased the results
A. Chalopin et al., 2017 [4]	CT	52 (19 F/33 M)	10.2	Fibular Hypoplasia	72 Tibias	Hexapod external fixation; Mono-lateral external fixator			47.4 months		Retrospective, group sizes were unequal.
S Riganti et al., 2018 [7]	CT	47 (19 F/25 M)	14.5	Idiopathic deformity of the lower limbs (group 1); congenital malformations of the lower limb (group 2)	Femurs (81.5% in group 1); Tibias and femurs (40% in group 2)	Hexapod external fixation (all)		Femur: 54.3; Tibia: 53.8	25.6 months	Pin site infections; Osteotomy translation; Delayed union; Compartment syndrome; Neuroapraxia; Pin breakage; Toes retraction; Knee subluxation	Small sample size
B. Blondel et al., 2009 [8]	CT	36 (11 F/25 M)	11.1	Congenital pathologies (17); Fractures (5); Post-traumatic sequelae (2); Post-infectious sequelae (3); Achondroplasia (3); Other pathologies (6)	Tibias (26); Femurs (6); other (4)	Hexapod external fixation (all)	4.3	38.2	6 years	Fracture of the regenerated bone (3); Superficial skin infection (8); Deep infection (1); Compartment syndrome (1); Transitory ankle equinus (2); Delayed union (2)	Small sample size
J. Horn et al., 2017 [9]	CT	192	14	Congenital deformities (group C) and acquired deformities (group A)	Tibias, Femurs	Hexapod external fixation (all)	3.9 (group C) 3.7 (group A)		32 months	Pin tract infections; fractures; Osteomyelitis; Compartment syndrome; Peroneal nerve entrapment; Subluxation of the knee	Small sample size
M. Fadel et al., 2004 [10]	RCT	22 (14 F/8 M)	16.5	Congenital short femur (3); Tibial shortening (5); Tibia vara (4); Genu valgum (2); Equinus foot (2); Posttraumatic fractures (6)		Hexapod external fixation (all)	5	42	3.2 years		Small sample size
P.L. Docquier et al. [11]	RCT	6	19.3	Infectious epiphysiodesis; Ollier’s disease; Idiopathic; Vitamin D-resistant; Hypophosphatemic rickets; Sequel of clubfoot and severe burn	Tibias (4); Femurs (1); Foot (2)	Hexapod external fixation (all)	3.6		16.2 months	Pin tract infections; Transient equinus deformity; Hamstrings retractions; Callus fracture; Non-union; Cavus deformity; Reflex sympathetic dystrophy	Small sample size

## Data Availability

Not applicable.
